# Variation in MHC genotypes in two populations of house sparrow (*Passer domesticus*) with different population histories

**DOI:** 10.1002/ece3.13

**Published:** 2011-10

**Authors:** Åsa Alexandra Borg, Sindre Andre Pedersen, Henrik Jensen, Helena Westerdahl

**Affiliations:** 1 Centre for Conservation Biology, Department of Biology, Norwegian University of Science and TechnologyTrondheim, Norway; 2 Molecular Ecology and Evolution Lab, Lund UniversityLund, Sweden

**Keywords:** Inbreeding, MHC variation, Microsatellites, *Passer domesticus*

## Abstract

Small populations are likely to have a low genetic ability for disease resistance due to loss of genetic variation through inbreeding and genetic drift. In vertebrates, the highest genetic diversity of the immune system is located at genes within the major histocompatibility complex (MHC). Interestingly, parasite-mediated selection is thought to potentially maintain variation at MHC loci even in populations that are monomorphic at other loci. Therefore, general loss of genetic variation in the genome may not necessarily be associated with low variation at MHC loci. We evaluated inter- and intrapopulation variation in MHC genotypes between an inbred (Aldra) and a relatively outbred population (Hestmannøy) of house sparrows (*Passer domesticus*) in a metapopulation at Helgeland, Norway. Genomic (gDNA) and transcribed (cDNA) alleles of functional MHC class I and IIB loci, along with neutral noncoding microsatellite markers, were analyzed to obtain relevant estimates of genetic variation. We found lower allelic richness in microsatellites in the inbred population, but high genetic variation in MHC class I and IIB loci in both populations. This suggests that also the inbred population could be under balancing selection to maintain genetic variation for pathogen resistance.

## Introduction

Genetic variation may determine the ability of a population to respond to environmental changes and hence significantly contribute to the persistence of a population (O'Brien et al. 1985; Sanjayan et al. 1996; Willi et al. 2006). Therefore, genetic variation is thought to be necessary for the long-term survival of populations and species (Frankham et al. 2002). This is especially important for small populations because they are more vulnerable to inbreeding (Saccheri et al. 1998; Westemeier et al. 1998; Madsen et al. 1999) and are also more likely to lose genetic variation through random genetic drift (Nei et al. 1975; Wright 1977; Lande 1988). In addition, bottleneck events may accelerate the loss of genetic variation (Hansson and Richardson 2005). Selection is also expected to affect intrapopulation levels of genetic variation. The presence of balancing selection may counteract the loss of variation due to drift, whereas strong directional selection is predicted to reduce variation and push alleles to fixation (Hughes and Yeager 1998; Lynch and Walsh 1998).

In vertebrates, the major histocompatibility complex (MHC) is one of the most variable regions of the genome (Garrigan and Hedrick 2003). The MHC genes encode cell-surface receptors that bind own and foreign peptides for presentation to T-cells (Hill et al. 1991; Janeway et al. 1999; Hedrick et al. 2001). Each MHC molecule can bind a limited number of peptides and hence it is important to be polymorphic at MHC loci to be able to resist a variety of pathogens. MHC molecules are divided into classes that differ in structure and function. MHC class I molecules interact with cytotoxic T cells for elimination of infected cells, and MHC class II molecules activate T helper cells that, for example, initiate antibody production (Janeway et al. 1999).

Low genetic variation at MHC has been associated with, for example, small population size (Újvari et al. 2002; Smulders et al. 2003) and potential inbreeding depression (Újvari et al. 2002). Balancing selection and point mutation in combination with gene duplication have been suggested to be the primary factors producing and maintaining MHC polymorphism (Nei et al. 1997; Garrigan and Hedrick 2003). Balancing selection includes overdominant selection, negative frequency dependent selection, as well as temporal and spatial variation in selection (Hedrick and Thomson 1983; Meyer and Thomson 2001; Hedrick 2002). Balancing selection can in all three forms be driven by pathogens. Overdominant selection favors MHC heterozygotes, which explains why natural populations show a deficiency in MHC homozygotes. Until recently, there was little evidence that heterozygote individuals actually are more resistant to disease (Apanius et al. 1997), but during the last decade, studies showing an effect of MHC heterozygosity on disease resistance have begun to accumulate (see e.g., Penn 2002; Sommer 2005; Evans and Neff 2009; Oliver et al. 2009). Alternatively, relatively rare alleles may be favored by selection through a close association between specific MHC genotypes and resistance to specific infectious diseases in a negative frequency dependent way. Finally, balancing selection could operate through spatial and/or temporal variation in selection pressure (Hedrick 2002). In all scenarios, general loss of genetic variation in the genome, for example due to inbreeding, may not necessarily be associated with a low variation at MHC loci (e.g., Richardson and Westerdahl 2003; Aguilar et al. 2004).

The house sparrow (*Passer domesticus*) is a small passerine bird with a wide natural distribution (Anderson 2006). When exploring adaptive significance of genetic variation, house sparrows are particularly interesting due to their success in colonizing most continents of the planet. This also makes them likely to be subject to variable selection pressures from pathogens (Loiseau et al. 2009). We will estimate inter- and intrapopulation variation in MHC class I and IIB genotypes as well as neutral markers in an inbred (Aldra) and a relatively outbred population (Hestmannøy) of house sparrows in a metapopulation at Helgeland, Norway. The two islands have markedly different population histories. The house sparrow population on Aldra was founded by one female and three males in 1998 after being extinct since the mid-1980s (Jensen et al. 2007). Immigration subsequent to the colonization event in 1998 may, however, have limited the genetic consequences of the strong bottleneck and founder event (Billing et al., unpubl. ms.). In contrast, the population size on Hestmannøy has been above 64 adult individuals since 1993 (H. Jensen and T. H. Ringsby, unpubl. ms.). Furthermore, reports from local contacts on the island confirmed that house sparrows had been present on Hestmannøy for many years prior to 1993 (Jensen et al., unpubl. ms.). Aldra is thus expected to have a lower genetic variation at neutral markers compared to Hestmannøy. Variation at MHC loci may also be expected to be lower unless there has been some sort of balancing selection acting on these loci. Both genomic DNA and transcribed (mRNA) MHC sequences will be analyzed and compared to microsatellite variation across a sample of individuals from the two populations.

## Materials and Methods

### Study species

The house sparrow is a Passerine bird that has a wide global distribution (Summers-Smith 1988; Anderson 2006). It is gregarious and sedentary, with low levels of natal dispersal (Summers-Smith 1988; Altwegg et al. 2000; Anderson 2006; Pärn et al. 2009). All year round, the house sparrow is closely associated with human settlement including urban areas, and often breeds and finds shelter and food inside farm buildings (Summers-Smith 1988; Anderson 2006).

Bonneaud et al. (2004) described sequences of both MHC class I and IIB in the house sparrow in two populations in France. In Bonneaud et al. (2004) genotyping seven individuals at exon 3 of MHC class I resulted in sequences of two different lengths, 20 different alleles, and a minimum of three loci in this gene family. For MHC class IIB, genotyping of the same individuals resulted in 13 alleles and a minimum number of three loci (Bonneaud et al. 2004).

### Study area and data collection

The two island populations included in this study were Aldra (66°24′N, 13°06′E) and Hestmannøy (66°32′N, 12°51′E) located approximately 13 km apart in an insular metapopulation off the coast of Helgeland in northern Norway (see map in Jensen et al. 2007). The islands in this metapopulation have been extensively studied on an individual-based level since 1993. Hence, nearly all adult birds on the study islands have been individually marked with a numbered metal ring and plastic color rings on their legs. Individual reproductive success has been determined by genetic analyses of neutral DNA markers from small blood samples collected from each individual bird (Jensen et al. 2003, 2004, 2008). Furthermore, observational and recapture data have given information on dispersal (Altwegg et al. 2000; Tufto et al. 2005; Pärn et al. 2009) and survival (Ringsby et al. 1998, 1999, 2002; Sæther et al. 1999; Jensen et al. 2004, 2007, 2008; Husby et al. 2006).

In early March 2007, blood was sampled by venipuncture from five randomly selected adult house sparrows in each of the two island populations. The birds were captured using mist nets placed inside cowsheds and barns on dairy farms. The population sizes on Aldra and Hestmannøy at that time were approximately 37 and 188 adult birds, respectively (H. Jensen, unpubl. data). From each sampled bird, we collected ca 25-µl blood, which was stored in 100% ethanol for later extraction of genomic DNA. In addition, ca 25-µl blood was collected and stored in TRIzol LS (Invitrogen, Life Technologies, Carlsbad, CA, USA), which maintains RNA in the sample without degradation. Samples were kept at –18°C until back in the laboratory, 0–2 days later, and were then stored at –80°C until analysis. All laboratory analyses were carried out at the Department of Biology, Norwegian University of Science and Technology, Norway.

### Isolation of DNA and RNA

Genomic DNA was isolated from whole blood samples according to the phenol–chloroform protocol described in Jensen et al. (2003). Total RNA was isolated from whole blood samples stored in TRIzol solution according to the manufacturer's protocol and treated with DNase (DNA-free; Ambion, Austin, TX, USA) to remove potential contamination of gDNA. Total cDNA was generated from the isolated RNA using the iScript™cDNA Sythesis Kit (Bio-Rad Laboratories, Hercules, CA, USA). The reverse transcription was done using approximately 1 µg of total RNA in a final volume of 20 µl and performed according to the manufacturer's instructions. RNA and DNA concentrations were determined using a NanoDrop ND-1000 spectrophotometer (NanoDrop Technologies, Wilmington, DE, USA).

### Microsatellite genotyping

To determine the intrapopulation levels of neutral genetic variation, we typed individuals from the two islands on 15 polymorphic microsatellite loci; Ase18 (Griffith et al. 2007), Pdoµ1 and Pdoµ3 (Neumann and Wetton 1996), Pdoµ5 (Griffith et al. 1999), Pdo10 (Griffith et al. 2007), Pdo16, Pdo17, Pdo19, Pdo22, Pdo27, Pdo32, Pdo33, Pdo40, Pdo44, and Pdo47 (Dawson et al., unpubl. ms.; Kekkonen et al. 2011). These loci are distributed across at least seven different chromosomes (Hansson et al. 2005; Dawson et al. 2006; Griffith et al. 2007; Dawson et al., unpubl. ms.), and thus likely to reflect the overall level of neutral genetic variation in the genome.

The forward primers were fluorescently labeled with either 6-FAM (Invitrogen), or VIC, NED, or PET (Applied Biosystems, Foster City, CA, USA), and a “pigtail” (i.e., GTT(T)-) was added to the 5′ end of the reverse primers. Seven and eight loci were multiplexed, respectively, in 10-µl polymerase chain reactions (PCRs) containing 5-µl QIAGEN Multiplex PCR Master Mix (QIAGEN, Düsseldorf, Germany), 2 µl of a mix of all primers (giving 0.063 µM final concentration of each primer in the 10-µl PCR reaction), and 3-µl genomic DNA. The PCR profile consisted of an initial denaturation step at 94°C (15 min), followed by 12 cycles where the temperature was decreased by 1°C each cycle starting at 62°C annealing temperature: 94°C (30 sec), 62–50°C (1.5 min), 72°C (1 min). These cycles were then followed by 25 cycles at 50°C annealing temperature: 94°C (30 sec), 50°C (1.5 min), 72°C (1 min). The PCR was ended by a final extension step at 60°C (5 min). After PCR, 0.5 µl of the multiplexed PCR products were mixed with 0.5-µl GeneScan LIZ600 (Applied Biosystems) and 10-µl HiDi Formamide (Applied Biosystems). Alleles were resolved in a 16 capillary ABI 3130xl Genetic Analyzer (Applied Biosystems) and scored using GeneMapper 4.0 (Applied Biosystems).

### MHC genotyping

The PCR reactions to amplify MHC alleles were done in a final volume of 25 µl, using 0.5 and 1 µg of gDNA and cDNA, respectively. The other ingredients in the reactions were 1× Buffer J (Invitrogen), MgCl_2_ (2.5 mM), primers (0.2 µM) (Pado1grw, 5′-TCCCCACAGGTYTCCACACMTG-3′, and A23H3 [Balakrishna et al. 2010] for class I; 2Zffw1 and 2zfrv1 [Balakrishna et al. 2010] for class IIB [Invitrogen]), dNTP's (2.5 mM, Invitrogen), 2.5 units of GoTaq®Flexi DNA Polymerase (Promega, Madison, WI, USA). The final concentrations of the reagents are listed within the brackets in the previous sentence. The primers used are degenerated and amplify most MHC class I (exon 3) and IIB (exon 2) alleles, respectively (H. Westerdahl pers. obs.). The PCR profile included an initial denaturation step at 94°C (5 min), followed by 35 amplification cycles at 94°C (1 min), 58°C (2 min), and 72°C (2 min), and a final extension step at 72°C (10 min). The PCR products were separated using 1.2% agarose gel electrophoresis and detected by ethidium bromide staining. The resulting bands (280 and 220 bp for class I and II, respectively) were cut out of the gel. The PCR product was purified from the gel plugs using QIAquick Gel Extraction Kit (QIAGEN). The purified PCR product was ligated into the pGEM®T Vector (Promega). Chemically competent cells INVαF’ (Invitrogen) were transformed with the ligated vector. Ten positive clones were selected using blue/white screening, grown overnight, and harvested for plasmid purification using a GenElute Plasmid Miniprep kit (Sigma-Aldrich, St. Louis, MO, USA). The purified plasmids were prepared for sequencing using the BigDye® Terminator v1.1 Cycle Sequencing Kit (Applied Biosystems) and M13 reverse primer, and sequenced using an ABI 3130xl Genetic Analyzer (Applied Biosystems). This procedure (from PCR to sequencing) was performed twice for each MHC primer on each individual, cDNA, and gDNA, respectively, to achieve the preferred number (i.e., 10) of readable sequences.

### Statistical analyses

Mean expected heterozygosity of microsatellite loci within each population was estimated using the observed allele frequencies and assuming unlinked loci and that the populations were in Hardy–Weinberg equilibrium (eq 8.4 in Nei 1987). Exact tests carried out in GenePop v. 4.0.10 showed that none of the loci deviated significantly from Hardy–Weinberg equilibrium either in the Hestmannøy population (*P* > 0.160) or in the Aldra population (*P* > 0.119), except for Pdoµ3, which had a level of significance of *P*= 0.024 in the Aldra population. After correcting for number of tests within the population (15; Bonferroni correction giving an α-level of 0.0033), this deviation from Hardy–Weinberg was however not significant. Tests for linkage disequilibrium (LD) carried out in GenePop v. 4.0.10 showed that none of the loci with enough information to carry out the test were in significant LD either within (*P* > 0.087) or across the two populations (*P* > 0.056). Moreover, microsatellite genotype data from a more extensive study that included the same loci except Pdo33 and Pdo40, but more sampled individuals from the two populations (Aldra *n*= 23 and Hestmannøy *n*= 43 (Jensen et al., in review)), showed that there after Bonferroni correction was only one locus (Pdo32; *P* < 0.001) that deviated from Hardy–Weinberg expectation in either population (all other loci *P* > 0.011). Furthermore, this extended microsatellite dataset showed that only three pairs of loci (Ase18–Pdoµ1, Pdoµ5–Pdo16, Ase18–Pdo36) in the Hestmannøy population and one pair of loci (Pdo10-Pdo17) in the Aldra population were in significant linkage disequilibrium with each other after adjusting the significance level for the large number of tests carried out within each population (Bonferroni-adjusted significance level: *P* < 0.00047). We used *t*-tests to examine whether the neutral genetic variation (i.e., average level of observed heterozygosity, mean expected heterozygosity, and allelic richness) differed between Aldra and Hestmannøy. Means are presented as ±1 standard error, and all statistical tests are two-tailed.

Sequences of MHC class I and IIB from the ABI 3130xl Genetic Analyzer (Applied Biosystems) were assembled and edited using BioEdit v. 7.0.0, MEGA v. 4.1, and aligned with MAFFT v. 6 online (http://align.bmr.kyushu-u.ac.jp/mafft/online/server/). Sequences were considered verified if they occurred in two independent PCRs. We further performed a BLAST search on GenBank (http://blast.ncbi.nlm.nih.gov/Blast.cgi), using our verified sequences, to look for published nucleotide sequences of MHC class I and IIB in house sparrows. These were used in the alignments and construction of trees. Because our primers are nested within those used by Bonneaud et al. (2004), our MHC class I sequences lack the first one or two bases compared to the sequences found in GenBank, after removing the primers on our sequences. This makes our sequences slightly shorter, which gives a reading frame error unless aligned with the GenBank sequences. The respective chicken sequences were used as outgroups (MHC class I: Gaga [AF013495], MHC class IIB: Gaga B-LB21 [AJ248585]). Neighbor-joining trees (supplemental material) were constructed using MEGA v. 4.1 with the maximum composite likelihood method including both transitions and transversions and bootstrapped 1000 times. Only bootstrapped trees were used. Selection for increased diversity in pathogen recognition is likely to be concentrated at sites involved in peptide presentation, and polymorphism has been found to be high in such regions (Hughes and Nei 1988; Parham et al. 1988; Hughes and Nei 1989). Peptide-binding region (PBR) codons are expected to be subject to positive selection, which promotes nonsynonymous substitutions, that in turn increase amino acid diversity of transcribed proteins (Hughes 2002). In non-PBR codons, purifying selection is thought to determine variation probably because of functional constraints of the protein. To determine putative PBR codons in the house sparrow, codons corresponding to PBRs in MHC class I and IIB in human MHC were superimposed upon the house sparrow sequences (Bjorkman et al. 1987; Brown et al. 1993). Evolutionary distances for synonymous and nonsynonymous substitutions of nucleotide sequences were calculated with the Nei–Gojobori (Jukes–Cantor) method using MEGA v. 4.1, computing overall means and standard errors with complete deletion of gaps/missing data, for PBRs and non-PBRs and for Aldra and Hestmannøy separately. These means were then used to calculate the ratio between nonsynonymous and synonymous substitutions (d_N_/d_S_). For trees and distances, rates were assumed to be uniform among sites, and patterns among lineages homogenous, and bootstrapped 1000 times to obtain SE values. We tested for positive selection (d_N_ > d_S_) on PBR and non-PBR regions of both MHC classes with the *Z*-test in MEGA v. 4.1, computing overall averages using the Nei–Gojobori (Jukes–Cantor) method. We also calculated codon-wise d_N_/d_S_ ratios and tested for positively selected amino acid sites with CODEML in the PAML 4 package (Yang 2007) using maximum likelihood models M1a (nearly neutral), M2a (positive selection), M7 (β), and M8 (β+ω), as well as in the online web-service of HyPhy (http://www.datamonkey.org/) using the REL method for site-by-site selection, the REV nucleotide substitution bias model, and the default significance level (Kosakovsky Pond et al. 2005a; b; Delport et al. 2010). Posterior probabilities for amino acid sites under positive selection were calculated using the Bayes Empirical Bayes method (Yang et al. 2005) in PAML, if models M2a and M8 fit significantly better than their neutral counterparts (M1a and M7, respectively) according to likelihood ratio tests (LRT). Nucleotide diversities were calculated using the program DnaSP v. 5. Average percent difference (APD, i.e., the average percentage of sequences that differ among individuals) was calculated according to Yuhki and O'Brien (1990). As in Bonneaud et al. (2004), we found MHC class I sequences of two different sizes (see results), these will from here on be denoted short and long sequences, respectively. Analyses of d_N_/d_S_ ratios on PBR and non-PBR sites, codon-wise d_N_/d_S_ ratios and positive selection, number of alleles per population, and nucleotide diversities have been performed on the two size categories separately. Statistical tests on MHC variation were performed with R v. 2.7.0 (R Development Core Team 2008) and SPSS v. 17.0.

## Results

### Neutral genetic variation

**Figure 1:**
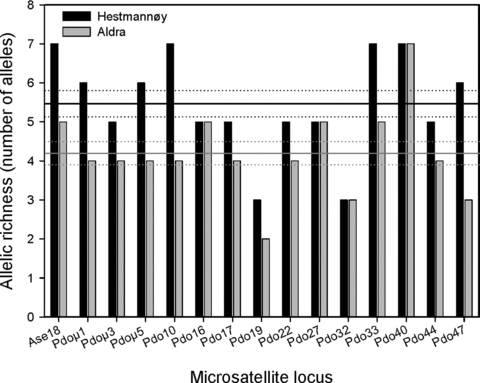
Allelic richness for each of the 15 microsatellite loci typed in house sparrows from Hestmannøy (black, *n*= 5) and Aldra (gray, *n*= 5). Horizontal lines give mean allelic richness (± 1 SE) across all 15 microsatellite loci for each population.

The mean allelic richness (i.e., average number of alleles) of the 15 microsatellite loci was significantly higher on Hestmannøy compared to Aldra (Fig. 1, independent samples *t*-test: *t*= 2.83, df = 28, *P*= 0.009). In fact, Aldra had a lower number of alleles in all but four microsatellite loci tested, the exceptions being Pdo16, Pdo27, Pdo32, and Pdo40, for which the numbers of alleles were the same for both populations. This resulted in a significant overall decrease in the number of alleles across loci on Aldra compared to Hestmannøy (Fig. 1, paired samples *t*-test: *t*= 4.75, df = 14, *P* < 0.001). In general, half or more of the alleles for each microsatellite locus were shared between the two populations, with one notable exception. For Pdo47, eight alleles were found in total, of which only one was shared, two were private to Aldra, and five to Hestmannøy. On average, Hestmannøy had significantly more private alleles than Aldra (Hestmannøy: 2.53 ± 0.27, Aldra: 1.20 ± 0.24; independent samples *t*-test, *t*= 3.64, df = 28, *P*= 0.001). There were however no significant differences between the two populations in mean observed heterozygosity (Hestmannøy: 0.79 ± 0.053, Aldra: 0.80 ± 0.052, independent samples *t*-test, *t*=–0.18, df = 28, *P*= 0.86), or mean expected heterozygosity (independent samples *t*-test, *t*= 1.57, df = 28, *P*= 0.13), although the mean expected heterozygosity was slightly higher on Hestmannøy (0.83 ± 0.053) than on Aldra (0.74 ± 0.033). The estimated neutral genetic differentiation (*F*_ST_) between the two populations based on microsatellite genotypes was 0.046.

### MHC variation

**Figure 2:**
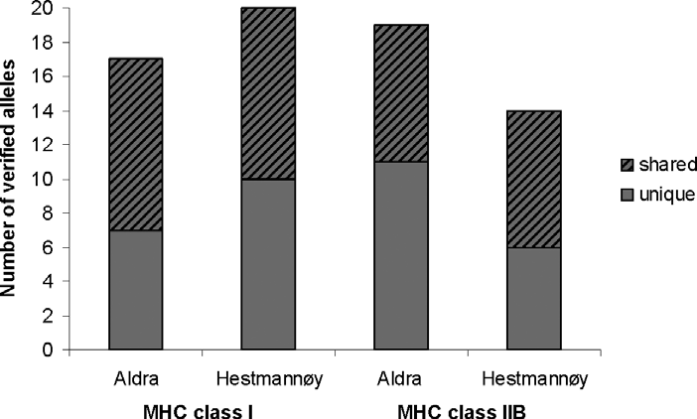
Number of MHC class I and IIB sequences found in two house sparrow populations differing in population history. Aldra is relatively inbred and recently went through a bottleneck and founder event. Bars show alleles private to each population (full) and those shared between the populations (striped).

In contrast to the results on microsatellites, variation at MHC loci, measured as total number of alleles, did not differ between the two populations (Fig. 2; Tables 1 and 2, χ^2^-test MHC class I: χ^2^= 0.111, df = 1, *P*= 0.739; MHC class IIB: χ^2^= 0.758, df = 1, *P*= 0.384). In addition, the average (mean ± SE) number of alleles per individual did not differ between the two populations in either MHC class I (Hestmannøy: 4.8 ± 0.58, Aldra: 5.2±0.58, Mann–Whitney U = 10, *P*= 0.575) or MHC class IIB (Hestmannøy: 4.2 ± 0.58, Aldra: 5.8 ± 0.58, Mann–Whitney U = 4.5, *P*= 0.09), although being slightly higher for MHC class IIB in Aldra. Consequently, for MHC class IIB, there was no difference in APD between Hestmannøy and Aldra (Hestmannøy: APD = 90.74 ± 2.87, Aldra: APD = 87.14 ± 2.00, Mann–Whitney U = 37.5, *P*= 0.34). However, APD did differ between the populations for MHC class I, being higher in Hestmannøy (Hestmannøy: APD = 95.34 ± 2.61, Aldra: APD = 88.04 ± 2.76, Mann–Whitney U = 24.5, *P*= 0.043). Nucleotide diversities for both MHC classes were similar for the two populations (MHC class I short: Hestmannøy π= 0.0246 [SD = 0.0018], Aldra π= 0.0226 [SD = 0.0019]; MHC class I long: Hestmannøy π= 0.119 [SD = 0.015], Aldra π= 0.123 [SD = 0.012]; MHC class II: Hestmannøy π= 0.217 [SD = 0.011], Aldra π= 0.217 [SD = 0.008]).

**Table 1:** Number of exon 3 class I MHC sequences found in individuals (labeled 1–10) from two different island populations of house sparrow in northern Norway. Stage and year of marking indicates whether the bird was juvenile (J) or adult (A) in the year it was first ringed, where year is given by two digits (e.g., 03 = 2003). Birds ringed as adults March 2007 (i.e., A07) were most likely recruits from the 2006 cohort (see e.g., Jensen et al. 2008). Blood samples (mRNA and gDNA) for MHC genotyping were collected in March 2007 for all individuals, thus all individuals were adults at the time of sampling. Sequences indicated in parentheses have been obtained from gDNA, while those without have been obtained from cDNA. The minimum number of loci within a given individual is determined from the total number of different sequences (gDNA and cDNA) in an individual divided by two. The letter after the sequence name indicates whether the sequence is 233 bp (S = short) or 237–240 bp (L = long) long

Population	Aldra					Hestmannøy						
				
Individuals Stage and year of marking Sequences	3 J03	10 J06	5 A05	6 A07	7 A06	2 J02	1 J98	8 A06	9 A07	4 J04	Number of individuals sharing allele	Population occurrence
Pado-UA*301 L							3(3)				1	Hestmannøy
Pado-UA*302 S									2		1	Hestmannøy
Pado-UA*303 S		2(2)		(1)				(1)	1		4	Both
Pado-UA*304 S (reading frame error)						4					1	Hestmannøy
Pado-UA*305 S (reading frame error)						(2)					1	Hestmannøy
Pado-UA*306 S (syn. with Pado-UA*307)	(3)				(1)					(1)	3	Both
Pado-UA*307 S (syn. with Pado-UA*306)									(2)		1	Hestmannøy
Pado-UA*308 S			(1)				(1)		3(1)		3	Both
Pado-UA*309 S	3(2)										1	Aldra
Pado-UA*310 S		(1)			2						2	Aldra
Pado-UA*311 S									(3)		1	Hestmannøy
Pado-UA*312 S			2(1)								1	Aldra
Pado-UA*313 S	1					(1)					2	Both
Pado-UA*314 L (syn. with Pado-UA*315)								2			1	Hestmannøy
Pado-UA*315 L (syn. with Pado-UA*314)		(1)	(2)	(6)	(3)			3(5)		3(3)	6	Both
Pado-UA*316 L										(3)	1	Hestmannøy
Pado-UA*317 L	3										1	Aldra
Pado-UA*318 L			2								1	Aldra
Pado-UA*319 L								3			1	Hestmannøy
Pado-UA*320 L			(1)		(2)			(2)		1(2)	4	Both
Pado-UA*321 L (syn with Pado-UA*322)		3									1	Aldra
Pado-UA*322 L (syn with Pado-UA*321)		1							3		2	Both
Pado-UA*323 L		2									1	Aldra
Pado-UA*324 S							(2)				1	Hestmannøy
Pado-UA*325 S		(2)		(1)		(2)					3	Both
Pado-UA*326 S	(1)								1		2	Both
Pado-UA*327 S	(1)			1			(1)		(1)		4	Both
Sequences per individual	14	14	9	9	8	9	10	16	17	13		
Expressed and genomic sequences	7(7)	8(6)	4(5)	1(8)	2(6)	4(5)	3(7)	8(8)	10(7)	4(9)		
Different sequences per population			17					20				
Different sequences per individual	6	7	5	4	4	4	4	5	8	4		
Minimum number of loci	3	4	3	2	2	2	2	3	4	2		

**Table 2:** Number of exon 2 class IIB MHC sequences found in individuals (labeled 1–10) from two different island populations of house sparrow in northern Norway. Stage and year of marking indicates whether the bird was juvenile (J) or adult (A) in the year it was first ringed, where year is given by two digits (e.g., 03 = 2003). Birds ringed as adults March 2007 (i.e., A07) were most likely recruits from the 2006 cohort (see e.g., Jensen et al. 2008). Blood samples (mRNA and gDNA) for MHC genotyping were collected in March 2007 for all individuals, thus all individuals were adults at the time of sampling. Sequences indicated in parentheses have been obtained from gDNA, while those without have been obtained from cDNA. The minimum number of loci within a given individual is determined from the total number of different sequences (gDNA and cDNA) in an individual divided by two

Population	Aldra					Hestmannøy						
				
Individuals Stage and year of marking Sequences	3 J03	10 J06	5 A05	6 A07	7 A06	2 J02	1 J98	8 A06	9 A07	4 J04	Number of individuals sharing allele	Population occurance
Pado-DAB*301 (syn. with Pado-DAB*302)							3(6)				1	Hestmannøy
Pado-DAB*302 (syn. with Pado-DAB*301)								2			1	Hestmannøy
Pado-DAB*303				3							1	Aldra
Pado-DAB*304			4(1)			1(3)					2	Both
Pado-DAB*305		(4)						5			2	Both
Pado-DAB*306	2								1(2)	(6)	3	Both
Pado-DAB*307		1			(1)						2	Aldra
Pado-DAB*308 (contains stop codon)				(3)							1	Aldra
Pado-DAB*309	2										1	Aldra
Pado-DAB*310					6(1)						1	Aldra
Pado-DAB*311			2(2)								1	Aldra
Pado-DAB*312			1		(2)						2	Aldra
Pado-DAB*313					2						1	Aldra
Pado-DAB*314							2		(3)		2	Hestmannøy
Pado-DAB*315		1	3	5		2	(1)				5	Both
Pado-DAB*316 (syn. with Pado-DAB*317)	3(4)				(3)				1(3)	4(2)	4	Both
Pado-DAB*317 (syn. with Pado-DAB*316)	(4)										1	Aldra
Pado-DAB*318	1(1)								4(1)		2	Both
Pado-DAB*319		4									1	Aldra
Pado-DAB*320						4(1)					1	Hestmannøy
Pado-DAB*321						(3)					1	Hestmannøy
Pado-DAB*322 (syn. with Pado-DAB*324)	1	(3)			(1)	2(1)	(2)	2(1)			6	Both
Pado-DAB*323									3		1	Hestmannøy
Pado-DAB*324 (syn. with Pado-DAB*322)		(2)									1	Aldra
Pado-DAB*325	1	(1)	(1)	(1)		(1)				(2)	6	Both
Sequences per individual	19	16	14	12	16	18	14	10	18	14		
Expressed and genomic sequences	10(9)	6(10)	10(4)	8(4)	8(8)	9(9)	5(9)	9(1)	9(9)	4(10)		
Different sequences per population			19					14				
Different sequences per individual	7	7	5	4	6	6	4	3	5	3		
Minimum number of loci	4	4	3	2	3	3	2	2	3	2		

**Figure 3:**
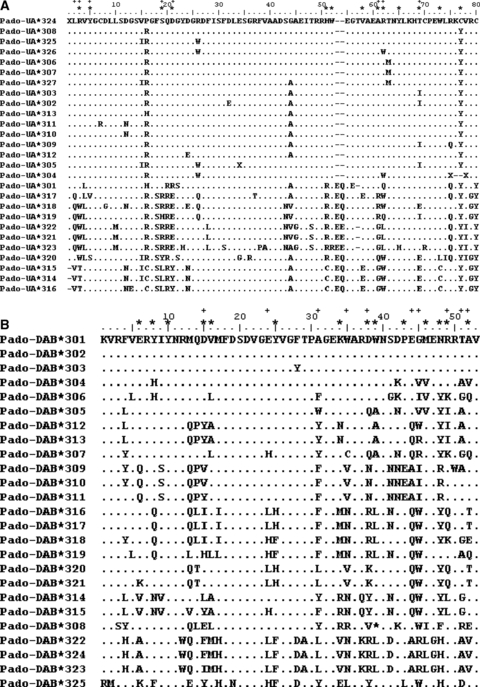
Translated amino acid sequences of the verified MHC class I (A) and class IIB (B) sequences. Dots (·) represent no difference between sequence and consensus/reference sequence, differences in amino acids are shown with their letter representation, gaps with minus signs (–), star (*) above the reference sequence represents putative peptide binding regions (PBRs) according to Bjorkman et al. (1987) and Brown et al. (1993) for MHC class I and IIB, respectively, while within a sequence the star represents a stop codon, the plus sign (+) represents codons under positive selection according to codon-wise analyses in PAML (for MHC class I only the long sequences had codons under positive selection according to PAML analyses), and unknown amino acids due to, for example, changes in reading frame after alignment are represented by X.

In total, we found 27 verified sequences of MHC class I exon 3, of which 20 (13 short and seven long) were found in Hestmannøy and 17 (10 short and seven long) in Aldra (Table 1). The two populations shared 10 MHC class I exon 3 sequences, which means that 10 and seven of the sequences were private to Hestmannøy and Aldra, respectively (Fig. 2). Of the sequences in Hestmannøy, two had a reading frame error (Pado-UA*304, Pado-UA*305, Figs. 3A and S1), both found within the same individual (Table 1). In addition, there were three sequence pairs that were found to be synonymous after translation to amino acid sequences, thus 22 different amino acid sequences and two nonfunctional amino acid sequences were found for MHC class I (Table 1).

For MHC class IIB exon 2, 25 verified sequences were found in total, distributed with 14 in Hestmannøy and 19 in Aldra (Table 2). Hestmannøy had six private sequences, while Aldra had 11, and eight sequences were shared by both populations (Fig. 2). One sequence found in Aldra contained a stop codon (Pado-DAB*308, Figs. 3B and S2). After amino acid sequence translation, three pairs of sequences were found to be synonymous (Table 1; Fig. S2). This resulted in 21 different MHC class IIB amino acid sequences and one nonfunctional amino acid sequence (Table 2).

Not all verified sequences were found as both genomic and transcribed sequences. Ten MHC class I sequences were found both in gDNA and cDNA samples, while eight were found only in gDNA and nine only in cDNA (Table 1). However, only seven MHC class I sequences were found in both gDNA and cDNA within the same individual. Of the 25 sequences of MHC class IIB, 15 were found in both gDNA and cDNA samples, four were found only in gDNA, and six only in cDNA (Table 2). Nine MHC class IIB sequences were found in both genomic and transcribed form within the same individuals.

The number of verified sequences found per individual varied between four and eight for MHC class I, and between three and seven for MHC class IIB, indicating a minimum of four loci for both MHC classes (Tables 1 and 2). All individuals had at least one transcribed (cDNA) sequence in both MHC classes (MHC class I: one to five transcribed sequences, MHC class IIB: one to six transcribed sequences, Tables 1 and 2). This gives a minimum of three transcribed loci for both classes of MHC.

As in previous studies on house sparrow MHC (Bonneaud et al. 2004), the MHC class I sequences found in this study could be further divided into two categories based on their length and alignment (Fig. S1). We found MHC class I sequences of two different lengths: 233 bp (16 sequences) and 237–240 bp (11 sequences) long. The shorter sequences all had a gap of 6 bp at 157 bp into the sequence, whereas about half of the longer sequences had a gap of 3 bp at 169 bp into the sequence. We amplified MHC class IIB sequences that were 159 bp long except in one case that had an extra C at the end of the sequence (Pado-DAB*316, Fig. S2). This translated into 78-80 and 53 amino acids for MHC class I and IIB sequences, respectively (Fig. 3). Trees (neighbor-joining, bootstrapped 1000 times) were constructed for both MHC classes with the sequences from Aldra and Hestmannøy, as well as the previously published sequences in GenBank. We found no segregation depending on population for either MHC class I or class IIB. Rather, several sequences from our study islands were closely related to sequences in GenBank (Figs. S3 and S4). Notably, one MHC class I sequence from Aldra (Pado-UA*318) was very similar to a sequence that has been found to be related to resistance to bird malaria in house sparrows (Pado-UA*123, Loiseau et al. 2008). The short MHC class I sequences formed a clear separate cluster, with a bootstrap value of 98, from the long MHC class I sequences in a neighbor-joining bootstrapped tree (Fig. S3).

PBRs of the MHC exons of both classes had consistently higher d_N_/d_S_ ratios than regions outside the PBRs (Table 3). However, only MHC class IIB PBRs in both populations showed significant signs of positive selection according to the *Z*-test, whereas both short and long MHC class I sequences in Aldra, as well as short MHC class I sequences in Hestmannøy, tended to do so (Table 3). Codon-wise selection analyses resulted in no sites under positive selection for MHC class I short sequences (all *P* > 0.11). However, eight (PAML) and six (HyPhy REL) sites were under positive selection for MHC class I long sequences, and 10 (PAML) and three (HyPhy REL) sites were under positive selection for MHC class IIB (Table 4). Codons under positive selection according to PAML analyses corresponded in seven cases (codons 3, 5, 19, 21, 61, 62, 69) to the PBR sites from Bjorkman et al. (1987) (Fig. 3A) for MHC class I long sequences, while for MHC class IIB, four positively selected codons (15, 38, 49, 52) corresponded to the PBR sites from Brown et al. (1993) (Fig. 3B).

**Table 3:** Mean and standard error of nonsynomymous (d_N_) and synonymous (d_S_) base substitutions as well as the ratio between them (d_N_/d_S_) in (A) MHC class I exon 3 and (B) MHC class IIB exon 2 sequences of two insular house sparrow populations in northern Norway. MHC class I sequences were further divided into two groups according to their lengths: short = 233 bp, long = 237–240 bp. d_N_/d_S_ ratios were calculated for overall distance (whole sequences), PBR and non-PBR, for each population separately. PBR represents the putative peptide binding coding regions of the exon, whereas non-PBR represents the rest of the exon. Distances of all sequences and overall average *Z*-tests were calculated using the Nei–Gojobori (Jukes–Cantor) method in MEGA v. 4.1, with complete deletion of missing data/gaps, homogenous pattern among lineages, uniform rates among sites. Errors of distances were bootstrapped 1000 times. nc = not computable

	Aldra				Hestmannøy			
		
	d_S_	d_N_	d_N_/d_S_	*Z*-test d_N_ > d_S_	d_S_	d_N_	d_N_/d_S_	*Z*-test d_N_ > d_S_
(A)								
MHC class I								
Short sequences								
PBR	0.000 ± 0.000	0.018 ± 0.013	nc	*Z*= 1.418	0.000 ± 0.000	0.020 ± 0.016	nc	*Z*= 1.300
				*P*= 0.079				*P*= 0.098
Non-PBR	0.059 ± 0.023	0.017 ± 0.006	0.29	*Z*=−1.736	0.066 ± 0.024	0.020 ± 0.008	0.30	*Z*=−1.848
				*P*= 1.000				*P*= 1.000
Long sequences								
PBR	0.234 ± 0.128	0.362 ± 0.091	1.55	*Z*= 1.346	0.429 ± 0.200	0.456 ± 0.157	1.06	*Z*= 0.179
				*P*= 0.090				*P*= 0.429
Non-PBR	0.172 ± 0.043	0.087 ± 0.015	0.51	*Z*=−1.973	0.133 ± 0.037	0.076 ± 0.016	0.57	*Z*=−1.462
				*P*= 1.000				*P*= 1.000
(B)								
MHC class IIB								
PBR	0.174 ± 0.068	0.523 ± 0.096	3.01	*Z*= 3.770	0.211 ± 0.087	0.483 ± 0.100	2.29	*Z*= 3.102
				*P* < 0.001				*P*= 0.001
Non-PBR	0.181 ± 0.053	0.198 ± 0.043	1.09	*Z*= 0.229	0.214 ± 0.062	0.208 ± 0.048	0.97	*Z*=−0.071
				*P*= 0.410				*P*= 1.000

**Table 4:** Codon-wise tests of positive selection on MHC class I and IIB sequences from two populations of house sparrows in northern Norway. MHC class I sequences were further divided into two groups according to their lengths: short = 233 bp, long = 237–240 bp. The model tests were performed with CODELM in the PAML 4 package. lnL_b_=log-likelihood value, 2ΔL=test value of likelihood ratio test (LRT), P=significance value

	N^1^	PAML				Positively selected sites^2^	
			
		Models	lnL_b_	2ΔL	*P*	PAML (BEB)^3^	REL^4^
MHC class I short	14	M1a (neutral)	−517.22			Not allowed	
		M2a (selection)	−516.91	0.63	0.73	-	
		M7 (β)	−517.29			Not allowed	
		M8 (β+ω)	−516.91	0.75	0.69	-	-
MHC class I long	11	M1a (neutral)	−951.52			Not allowed	
		M2a (selection)	−933.06	36.93	<0.0001	3**, 5*, 19**, 21**, 61**, 62**, 69**	
		M7 (β)	−951.78			Not allowed	
		M8 (β+ω)	−932.98	37.61	<0.0001	2*, 3**, 5*, 19***, 21**, 61***, 62***, 69***	3*, 19*, 21*, 61*, 62**, 69**
MHC class IIB	24	M1a (neutral)	−1344.49			Not allowed	
		M2a (selection)	−1307.29	74.42	<0.0001	15**, 24**, 31**, 38*, 44**, 45**, 49**, 51**, 52*	
		M7 (β)	−1346.38			Not allowed	
		M8 (β+ω)	−1309.58	73.59	<0.0001	15***, 24***, 31***, 35**, 38**, 44***, 45***, 49***, 51***, 52**	31**, 44**, 51**

1 number of sequences, excluding sequences containing reading frame error (*N*= 2 MHC class I short) or stop codon (*N*= 1 MHC class IIB).

2 significance levels for selected sites *≤0.05, **≤0.01, ***≤0.001.

3 calculated using the Bayes Empirical Bayes (BEB) method (Yang et al. 2005).

4 computed in HyPhy online service (http://www.datamonkey.org/) using the REL method for site-by-site selection, the REV nucleotide substitution bias model, and the default significance level, for comparison given in the same row as PAML M8 sites.

## Discussion

We estimated variation at neutral (microsatellite) and adaptive (MHC) loci in two house sparrow populations differing in recent population history. One population (Aldra) was established 10 years prior to sampling by a handful of individuals, while the other population (Hestmannøy) had been a comparably large population for decades. As predicted, we found a lower level of genetic variation in neutral markers in Aldra compared to Hestmannøy (Fig. 1). In contrast, the genetic variation at MHC loci was equally high in both populations (Fig. 2; Tables 1 and 2).

The results on microsatellite variation are in accordance with Jensen et al. (2007), which showed that Aldra had a higher average level of inbreeding than Hestmannøy, as well as a lower level of heterozygosity. This was expected because the two populations have different histories. The population on Aldra was founded by one female and three males in 1998. During the subsequent years, the population size increased fast, until it leveled out at approximately 35–50 individuals from 2003 and onwards (Billing et al., unpubl. ms.). Jensen et al. (2007) also showed an association between a higher inbreeding coefficient and lower heterozygosity among individuals in the Helgeland island metapopulation (including Aldra and Hestmannøy). Furthermore, inbreeding had negative fitness consequences in these populations through reduced recruitment probability of fledglings (Jensen et al. 2007). In light of the previous results in the same study populations, lower genetic variation was also expected at MHC loci on Aldra. This was, however, not the case (Tables 1 and 2). Instead, we found similar numbers of MHC class I and IIB sequences in the two populations. The populations in our study also shared a large proportion of the sequences of both MHC classes, while about half of the sequences found in a specific population were private to that population (Fig. 2; Tables 1 and 2). It is, however, likely that we would have found different numbers of private and shared sequences with a larger sample size.

In light of the results from our microsatellite data, it may seem surprising that a small inbred population that recently went through a founder event had comparable levels of genetic variation at MHC loci to a relatively large and stable population in the same area. This is also because theoretical population genetics models predict a strong negative relationship between population size and the rate of loss of genetic variation through random genetic drift (e.g., Wright 1969). Consequently, genetic drift should have resulted in a higher loss of MHC alleles in the relatively small Aldra population compared to the larger Hestmannøy population. In the endangered and highly inbred New Zealand Robin (*Petroica traversi*), for example, drift seems to have resulted in monomorphism in at least one class of MHC loci (Miller and Lambert 2004). Similarly, an island population of a threatened tuatara reptile (*Sphenodon guntheri*) had a substantially lower level of MHC variation than a nearby large population of a related species (*S*. *punctatus*, Miller et al. 2008). Miller et al. (2008) suggested that this indicated a recent population bottleneck in *S. guntheri*. However, since MHC genes are fitness-related and thought to be under balancing selection, variation may be more likely to be maintained even in populations that have lost variation at other loci. An example is the San Nicolas Island fox (*Urocyon littoralis dickeyi*), which is extraordinarily monomorphic in hypervariable neutral genetic markers, but shows unexpectedly high level of heterozygosity in four MHC class II loci (Aguilar et al. 2004). By simulation, the authors showed that this pattern could arise in a bottlenecked population subject to intense balancing selection.

One could speculate about the mechanisms behind the selection for maintaining MHC diversity in a population such as Aldra. Balancing selection could come about through interactions with pathogens, or possibly inbreeding avoidance mechanisms such as mate choice. A study by Engen et al. (2007) showed that Aldra had higher growth rate and ratio of effective population size to actual population size (N_e_/N), as well as a lower total demographic variance compared to Hestmannøy. Thus, a larger proportion of the adult population on Aldra reproduce compared to on Hestmannøy. This could reduce the rate of allele loss on Aldra. In addition, the most likely mating pattern on Aldra seems to be random mating (Billing et al., unpubl. ms.). Because Aldra was founded by only four individuals, among them one female, and the population size thereafter has been relatively low, the potential for mate choice could have been limited. The high MHC variation in this population may also be a result of gain rather than reduced loss of alleles. New alleles could have been introduced by immigration, after the founder event, if the immigrants reproduced successfully. In the first 10 years, after the Aldra population was established, 15 immigrants were recorded (Billing et al., unpubl. ms.) and these individuals are likely to have carried previously lost or novel alleles. Analyses have shown that these immigrants also contributed to the population's gene pool (Billing et al., unpubl. ms.). There may also be selection against inbred individuals on Aldra, since lifetime reproductive success was negatively related to individual inbreeding level in this population (Billing et al., unpubl. ms.), although the mechanisms behind this selection are not known yet.

Finally, selection pressure caused by exposure to a variable community of pathogens may have helped to maintain the MHC variation gained or restored by immigration. For example, in insular avian populations of the West Indies, avian malaria communities have been shown to be relatively stable over short time periods (1 year) but to have lineage turnover during the course of a decade (Fallon et al. 2004). Infection of different lineages of avian malaria in willow warblers (*Phylloscopus trochilus*) in Sweden showed both temporal and geographical variation between three different sampling years (Bench and Åkesson 2003). In particular, two species of *Haemoproteus* seemed to have almost replaced each other, one first being prominent in southern Sweden and then in the north, while the other species had more or less the opposite pattern. Thus, over a short time period selective pressures from pathogens may be similar on a local geographical scale, but over a longer time period geographically more distant populations may also encounter similar pathogen lineages. Therefore, selection may have promoted genetic variation at MHC loci also in the Aldra population in spite of possibly stronger genetic drift than in the Hestmannøy population. In line with this, PBRs of MHC class IIB sequences from both populations showed signs of positive selection. Several parasites infect house sparrows in this insular metapopulation at Helgeland in northern Norway, such as avian *Hippoboscidae* (Ringsby et al., unpubl. data), feather mites (*Ornithonyssus* spp., Jensen and Pärn, unpubl. data), *Isospora* spp. and *Capillaria spp.* (Holand et al., unpubl. data), as well as the parasitic gapeworm *Syngamus trachea* (Holand et al., unpubl. ms.). House sparrows infected with *S. trachea* have been found on both Aldra and Hestmannøy, and individuals showing symptoms of gapeworm infection have a reduced probability of survival until the next breeding season (Holand et al., unpubl. ms.). Thus, similar selection pressure, such as exposure to the same pathogens, is one possible mechanism that should be explored in relation to maintenance of MHC variation in future studies.

## Conclusions

Variation at neutral loci may not reflect variation at adaptive loci. This was clearly demonstrated in the current study where we found high levels of MHC variation in an inbred house sparrow population with a low level of variation at neutral loci. The level of MHC variation in the inbred population was also comparable to that of a much larger outbred house sparrow population in the same area. The mechanisms behind such maintained functional genetic variation need to be further investigated, but immigration and selection on MHC variation through pathogens are possible candidates in this system.

## Data Accessibility

-DNA sequences: Genbank accessions:

Pado-UA*324 JN609623

Pado-UA*308 JN609624

Pado-UA*325 JN609625

Pado-UA*326 JN609626

Pado-UA*306 JN609627

Pado-UA*307 JN609628

Pado-UA*327 JN609629

Pado-UA*303 JN609630

Pado-UA*302 JN609631

Pado-UA*313 JN609632

Pado-UA*311 JN609633

Pado-UA*310 JN609634

Pado-UA*309 JN609635

Pado-UA*312 JN609636

Pado-UA*305 JN609637

Pado-UA*304 JN609638

Pado-UA*301 JN609639

Pado-UA*317 JN609640

Pado-UA*318 JN609641

Pado-UA*319 JN609642

Pado-UA*322 JN609643

Pado-UA*321 JN609644

Pado-UA*323 JN609645

Pado-UA*320 JN609646

Pado-UA*315 JN609647

Pado-UA*314 JN609648

Pado-UA*316 JN609649

## Supporting Information

**Figure S1.** Aligned nucleotide sequences of verified MHC class I sequences in two house sparrow populations at Helgeland, Norway.

**Figure S2.** Aligned nucleotide sequences of verified MHC class IIB sequences in two house sparrow populations at Helgeland, Norway.

**Figure S3.** Neighbor-joining bootstrapped (1000 times) tree of verified MHC class I sequences in two house sparrow populations at Helgeland, Norway.

**Figure S4.** Neighbor-joining bootstrapped (1000 times) tree of verified MHC class IIB sequences in two house sparrow populations at Helgeland, Norway.
