# Supplementary figures and images for: Variation in MHC genotypes in two populations of house sparrow (Passer domesticus) with different population histories
Source: Ecol Evol. 2011 Oct;1(2):145–59. doi: 10.1002/ece3.13 (PMC3287304; doi:10.1002/ece3.13)

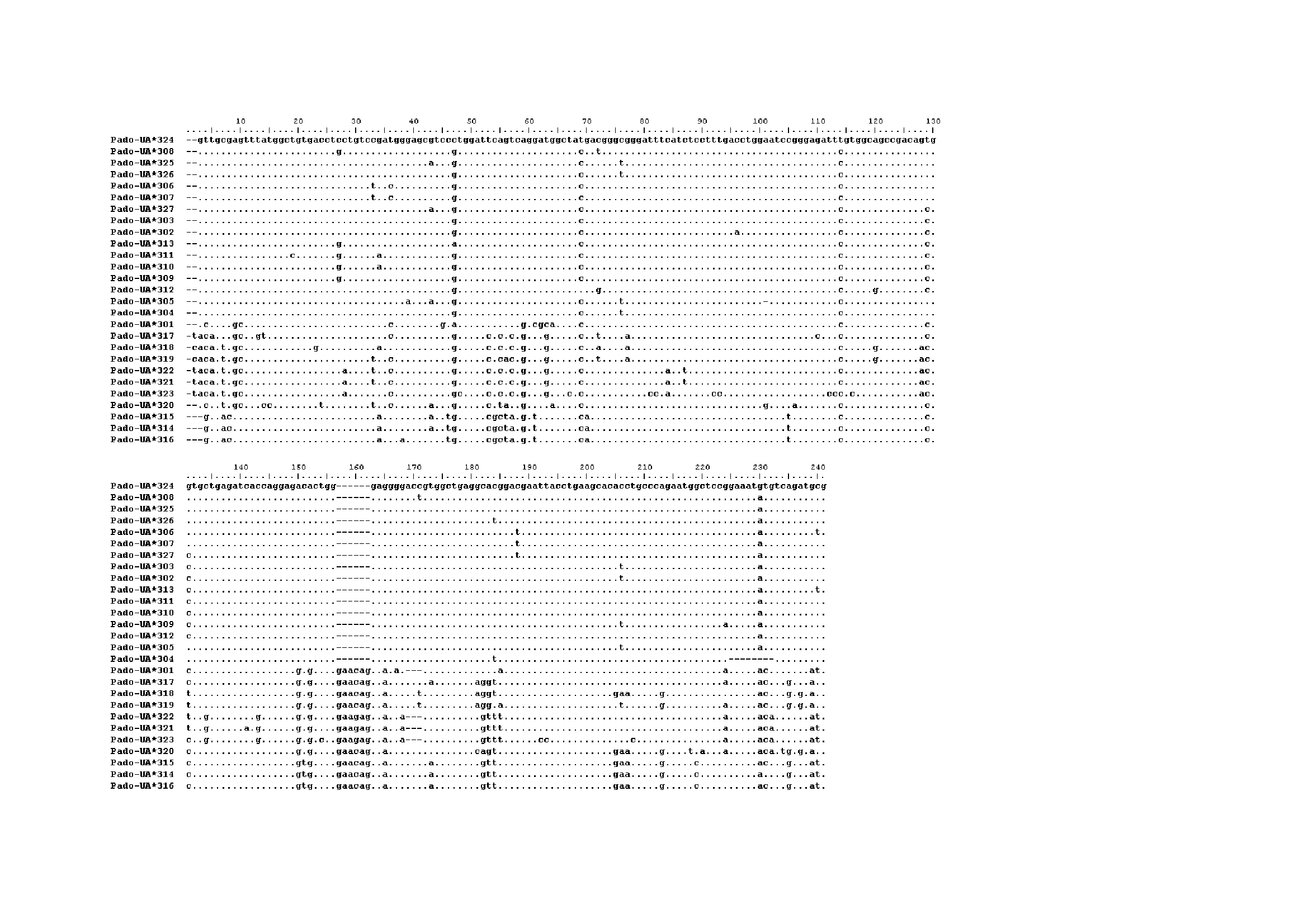

Supplement: Supplementary file 1 [file ece30001-0145-SD1.tif]

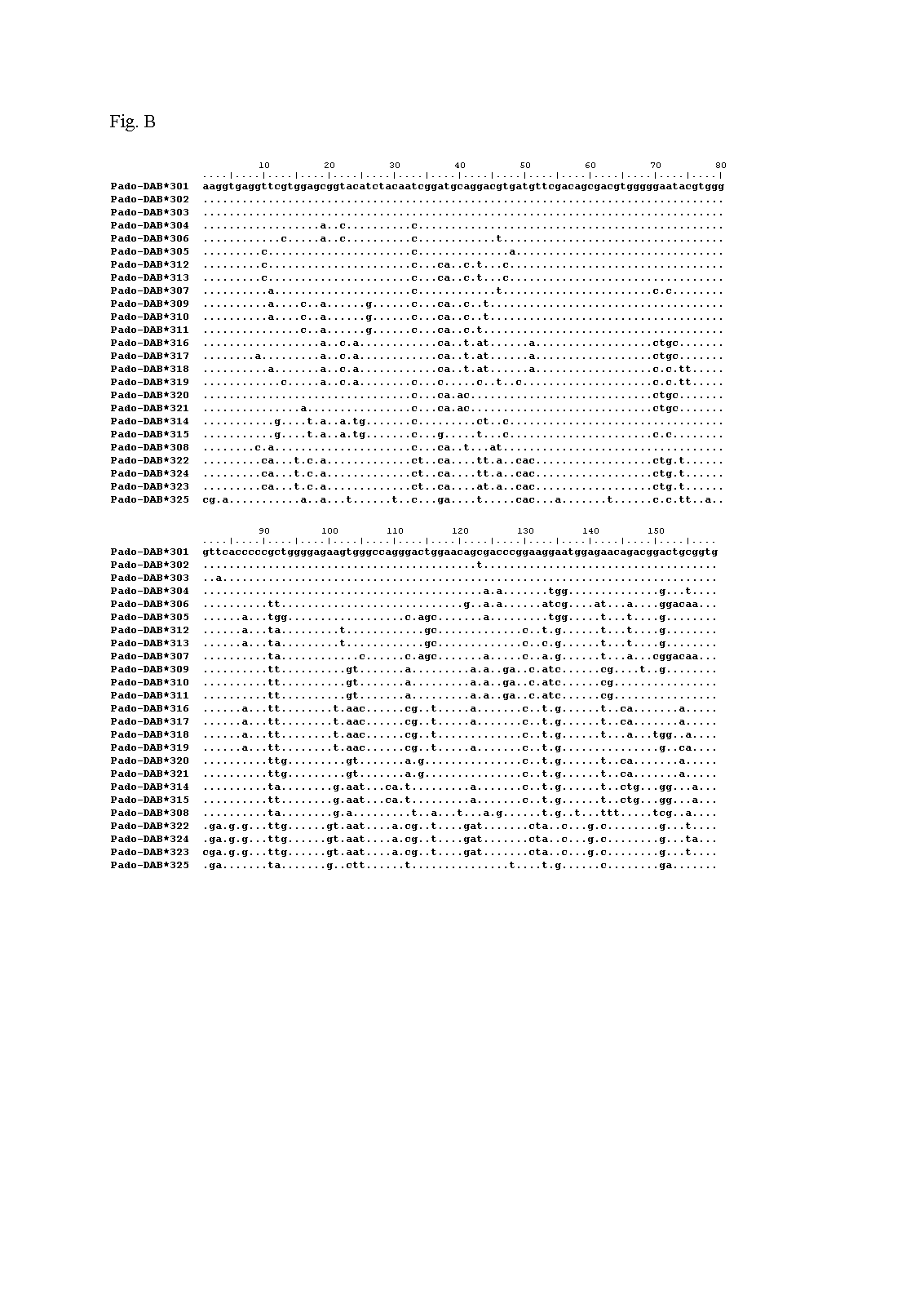

Supplement: Supplementary file 2 [file ece30001-0145-SD2.tif]

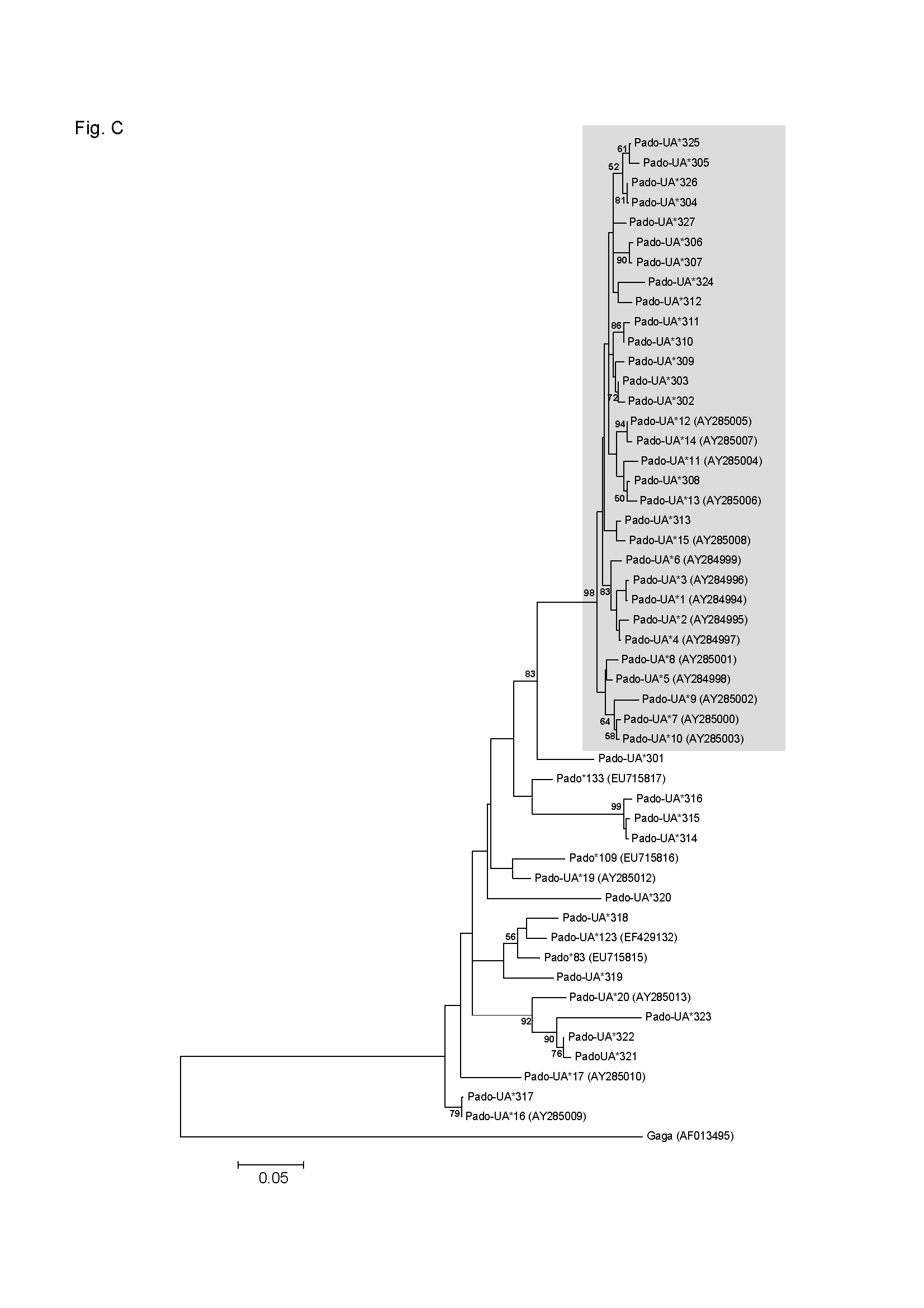

Supplement: Supplementary file 3 [file ece30001-0145-SD3.tif]
