# Supplementary material for: Variation in MHC genotypes in two populations of house sparrow (Passer domesticus) with different population histories
Source: Ecol Evol. 2011 Oct;1(2):145–59. doi: 10.1002/ece3.13 (PMC3287304; doi:10.1002/ece3.13)
Supplement: Supplementary file 5 [file ece30001-0145-SD5.doc]

**Supporting information**

Supporting figure legends

Fig. A. Aligned nucleotide sequences of verified MHC class I sequences in two house sparrow populations at Helgeland, Norway. Positions with the same base as the consensus/reference sequence are shown as dots (·), differing bases are shown with their letter representation (a, t, c, g) and gaps after alignment with a minus sign (-).

Fig. B. Aligned nucleotide sequences of verified MHC class IIB sequences in two house sparrow populations at Helgeland, Norway. Positions with the same base as the consensus/reference sequence are shown as dots (·), differing bases are shown with their letter representation (a, t, c, g) and gaps after alignment with a minus sign (-).

Fig. C. Neighbour-joining bootstrapped (1000 times) tree of verified MHC class I sequences in two house sparrow populations at Helgeland, Norway. The tree shows the relation between the sequences found in this study and previously published sequences found on GenBank. Chicken MHC class I sequence (Gaga (AF013495)) is used as outgroup. Only boostrap values above 50 are shown. The cluster of short MHC I sequences is shaded in grey.

Fig. D. Neighbour-joining bootstrapped (1000 times) tree of verified MHC class IIB sequences in two house sparrow populations at Helgeland, Norway. The tree shows the relation between the sequences found in this study and previously published sequences found on GenBank. Chicken MHC class II sequence (Gaga (B-LB21)) is used as outgroup. Only boostrap values above 50 are shown.
